# Ultrafine Co-Species Interspersed g-C_3_N_4_ Nanosheets and Graphene as an Efficient Polysulfide Barrier to Enable High Performance Li-S Batteries

**DOI:** 10.3390/molecules28020588

**Published:** 2023-01-06

**Authors:** Shanxing Wang, Xinye Liu, Yuanfu Deng

**Affiliations:** 1 School of Chemistry and Materials Engineering, Huizhou University, Huizhou 516007, China; 2 The Key Laboratory of Fuel Cell Technology of Guangdong Province, School of Chemistry and Chemical Engineering, South China University of Technology, Guangzhou 510640, China; 3 Electrochemical Energy Engineering Research Center of Guangdong Province, South China University of Technology, Guangzhou 510640, China

**Keywords:** co-species nanodots, g-C_3_N_4_ and graphene, polysulfide barrier, chemical adsorption, catalytic conversion

## Abstract

Lithium-sulfur (Li-S) batteries are regarded as one of the promising advanced energy storage systems due to their ultrahigh capacity and energy density. However, their practical applications are still hindered by the serious shuttle effect and sluggish reaction kinetics of soluble lithium polysulfides. Herein, g-C_3_N_4_ nanosheets and graphene decorated with an ultrafine Co-species nanodot heterostructure (Co@g-C_3_N_4_/G) as separator coatings were designed following a facile approach. Such an interlayer can not only enable effective polysulfide affinity through the physical barrier and chemical binding but also simultaneously have a catalytic effect on polysulfide conversion. Because of these superior merits, the Li-S cells assembled with Co@g-C_3_N_4_/G-PP separators matched with the S/KB composites (up to ~70 wt% sulfur in the final cathode) exhibit excellent rate capability and good cyclic stability. A high specific capacity of ~860 mAh g^−1^ at 2.0 C as well as a capacity-fading rate of only ~0.035% per cycle over 350 cycles at 0.5 C can be achieved. This bifunctional separator can even endow a Li-S cell at a low current density to exhibit excellent cycling capability, with a capacity retention rate of ~88.4% at 0.2 C over 250 cycles. Furthermore, a Li-S cell with a Co@g-C_3_N_4_/G-PP separator possesses a stable specific capacity of 785 mAh g^−1^ at 0.2 C after 150 cycles and a superior capacity retention rate of ~84.6% with a high sulfur loading of ~3.0 mg cm^−2^. This effective polysulfide-confined separator holds good promise for promoting the further development of high-energy-density Li-S batteries.

## 1. Introduction

With the sustained growth of energy density requirements in advanced portable electronic devices and electric vehicles, the development of a new generation of secondary batteries with high energy density is extremely demanded [1,2,3]. As a strong competitor, lithium-sulfur (Li-S) batteries have attracted tremendous attention due to their ultrahigh energy density of ~2600 Wh kg^−1^, and elemental sulfur also possesses multiple advantages of low cost, rich availability, and environmental friendliness [4]. Nevertheless, the practical applications of Li-S cells currently face several intrinsic challenges, such as the notorious shuttle effect and sluggish reaction kinetics of intermediate polysulfides. The shuttle effect is caused by the diffusion of polysulfides dissolved in electrolytes through the separator, which usually leads to irreversible capacity attenuation and poor Coulombic efficiency [5,6]. Even worse, the sluggish redox kinetics originated from the inherent multi-step solid–liquid–solid heterogeneous reaction mechanism inevitably begets the accumulation of various sulfur species instead of immediate conversion and then causes more diffusion behavior of polysulfides, ultimately resulting in low sulfur use and a short cycling lifespan [7]. 

To address the aforementioned issues, extensive efforts have been devoted to mitigate the intrinsic drawbacks. One of the most effective strategies is to design functional conductive carbonaceous materials for capturing sulfur/polysulfides, such as tuning the pore size and/or porosity (micropore, mesopore, etc.) [7,8,9], doping various heteroatoms (N, O, S, P, etc.) [10,11,12,13], and tailoring the morphology (CNTs, graphene, etc.) [14,15], which indeed achieves a positive effect on boosting the performance of Li-S cells, owing to the physical confinement or chemical adsorption of carbonaceous substances toward polysulfides. Nonetheless, the shuttle behavior of intrinsic soluble polysulfides in electrolytes can be only partially restricted because of the limited absorbability originated from the functional carbons for dissolved polysulfides, leading to unsatisfied cyclic stability over long-term cycling [16,17]. In addition, it can cause the accumulation of sulfur species due to their sluggish redox conversion kinetics on the carbonaceous matrixes, which thus easily results in the diffusion of intercepted polysulfides along cycling [16,18,19]. Impressively, it has been indicated that some metal-based additives introduced into carbon substrates play an important role in the chemical adsorption and/or catalytic conversion of intermediate polysulfides, such as metal oxides (Ti_4_O_7_, Nb_2_O_5_, etc.) [20,21], metal sulfides (CoS_2_, MoS_2_, etc.) [22,23], metal nitrides (MoN, VN, etc. [18,24]), and metal nanoparticles (Fe, Co, Ni, etc.) [16,25,26], synergistically expediting the reaction kinetics of polysulfides in the redox process. Unfortunately, the chemical interaction between sulfur species and some inorganics usually can be influenced significantly by the dimension and other physical nature of the inorganic additives, thus leading to the formation of a thick passivation layer on the surface of inorganics, especially under high sulfur areal loading and long-term cycling [27,28], which finally has an adverse impact on the performance of Li-S cells under long-term cycling conditions. Therefore, it is of great importance to elaborate suitable metal-based additives with chemisorption polysulfides and timely accelerating their conversion to Li_2_S during the redox reaction.

Specifically, recent studies have demonstrated that tailoring metal compounds to an ultra-small size, such as nanocrystals, nanodots, and clusters, and even to the single-atomic scale could generate strong adsorption and excellent catalytic activity for polysulfides, owing to enhanced surface energy and more active terminations [16,29,30,31]. However, it is a great challenge to fabricate highly dispersed ultrafine metal compounds because the isolated ultra-small particles with high surface energy can susceptibly agglomerate into big particles in the synthesis process. Various strategies have been explored to minimize the agglomeration of isolated ultrafine particles; to this end, the most common approach is to restrict the further growth of nanocrystals by functional support and pore size confinement [32,33,34,35]. For example, Liu et al. designed highly dispersed TiO_2_ nanoparticles anchored onto Co-nanoparticle-decorated carbon polyhedras derived from ZIF-67 (C-Co/TiO_2_) as a sulfur host. The as-prepared S@C-Co/TiO_2_ cathode exhibited enhanced discharge capacity and long cycling capability due to the stronger chemisorption of C-Co/TiO_2_ for polysulfides [35]. Recently, Zhang et al. successfully fabricated MoP-quantum-dot-modified N, P-carbon nanotubes (MPQ@NP-HCNT) as a multifunctional modification layer for the Li-S separator. As a result, MoP quantum dots had a large adsorption energy for S_8_ and Li_2_S_n_ and improved the kinetics of the subsequent Li_2_S reaction simultaneously [36]. Such impressive results can give some guidance to rationally design an ultra-small-size adsorber/catalyst for polysulfides within carbonaceous matrix composites. However, there are some drawbacks, especially in the preparation of precursors, such as expensive reagents, harsh reaction conditions, and synthetic complexity, which have become a major hindrance to their large-scale commercial applications. Consequently, designing a feasible synthesis strategy with advantages of a simple procedure and low cost for fabrication of new ultra-small-size metal compounds coupled with a carbon matrix is urgently demanded.

Considering these issues, herein, we successfully fabricated highly dispersed ultrafine-Co-species-nanodot-interspersed g-C_3_N_4_ nanosheets and graphene composites (Co@g-C_3_N_4_/G) as separator coating materials using a facile and cost-effective method—pyrolysis of a mixed precursor consisting of g-C_3_N_4_, CoCl_2_·6H_2_O, and graphene oxide (GO) mixture. In this design, g-C_3_N_4_ could partially decompose to realize the reduction of GO, while serving as a template to leave some pores during calcination treatment [37,38]. Within the Co@g-C_3_N_4_/G products, the residual g-C_3_N_4_, graphene, and ultrafine Co-species could form a unique heterostructure attributed to the intimate interface interactions between graphene and g-C_3_N_4_, both with the 2D structure of nanosheets [39,40]. Hence, graphene here acted as an ideal platform for electron transfer between the residual g-C_3_N_4_ and the catalytic Co-species in the heterostructure. This heterostructural composite as a separator-modified layer can not only enable effective chemisorption toward polysulfides but also catalyze the conversion of intercepted polysulfides. As a result, the assembled Li-S cells using Co@g-C_3_N_4_/G-PP separators paired with a high-sulfur-content electrode (~70 wt%) exhibit excellent rate capability and good cyclic stability. A high specific capacity of ~860 mAh g^−1^ at 2.0 C as well as a capacity-fading rate of only ~0.035% per cycle over 350 cycles at 0.5 C can be achieved. This bifunctional separator can even endow a Li-S cell at a low current density to exhibit excellent cycling capability, with a capacity retention rate of ~88.4% at 0.2 C over 250 cycles. Furthermore, a Li-S cell with a Co@g-C_3_N_4_/G-PP separator possesses a stable specific capacity of 785 mAh g^−1^ at 0.2 C after 150 cycles and a superior capacity retention rate of ~84.6% with a high sulfur loading of ~3.0 mg cm^−2^.

## 2. Results and Discussion

### 2.1. Synthesis and Characterization of the Co@g-C_3_N_4_/G Heterostructure Composites

**Scheme 1:**
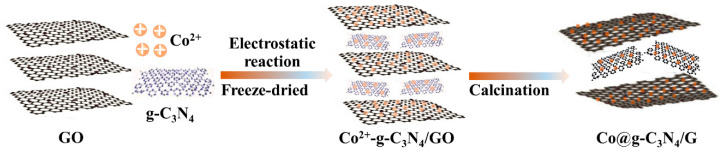
Schematic diagram of the preparation process of the Co@g-C_3_N_4_/G heterostructure.

g-C_3_N_4_ was fabricated using a simple and low-cost strategy according to previous work. A schematic illustration of the synthesis process of Co@g-C_3_N_4_/G is shown in Scheme 1, that is, CoCl_2_·6H_2_O was dispersed into the GO suspensions, accompanied by the addition of as-prepared g-C_3_N_4_ by vigorous sonication to obtain a homogeneous precursor solution. As a result, the cobalt cations could be dispersed uniformly, enabled by the dual-anchoring effect of g-C_3_N_4_ and GO. This phenomenon can be attributed to the following two major aspects: (1) It has been authenticated that 2D g-C_3_N_4_ nanosheets can be as an ideal platform to trap transition metal/metal ions firmly on account of its abundant polygonal cavities and high-density N atoms, as well as electronegativity [41,42], and (2) the rich oxygen-containing functional groups (-OH, -COOH, etc.) grafted on the surface of graphene oxide could also in situ anchor metal cations under an aqueous solution state via the electrostatic force or coordination [43], namely implying that GO nanosheets can behave as a surfactant and thus uniformly disperse the anchored Co^2+^ ions effectively [44]. As expected, highly dispersed ultrafine-Co-species-nanodot-interspersed g-C_3_N_4_ nanosheets and graphene heterostructure nanocomposites were obtained eventually after calcination treatment. 

**Figure 1:**
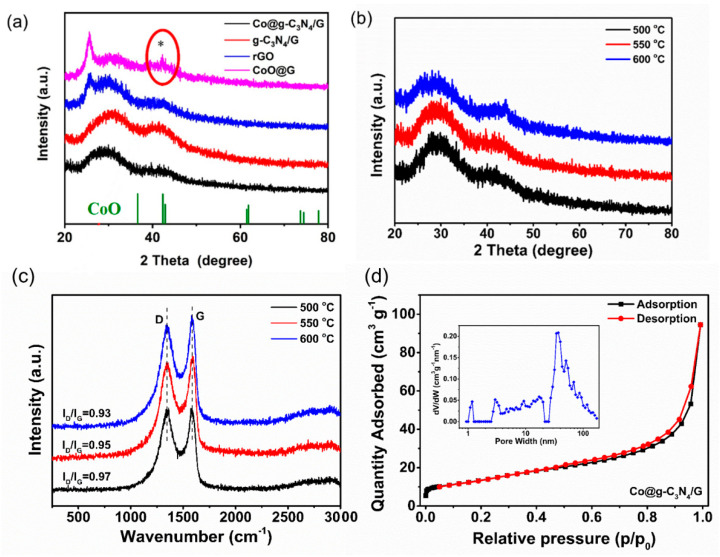
(**a**,**b**) XRD spectra, (**c**) Raman spectra, and (**d**) N_2_ adsorption–desorption curve of as-prepared samples (the corresponding pore size distribution curves are inserted in (**d**)). The * in Figure 1a is a diffraction peak of CoO in the CoO@G sample.

The crystal structures of as-prepared samples were investigated using XRD, as shown in Figure 1a. A characteristic peak was observed at 26.5° in both CoO@G and G samples, and this peak was assigned to the C (002) diffraction peak [45]. Besides, the CoO@G sample had an extra peak at 42.4°, corresponding to the (200) crystal facet of CoO (JCPDS No. 75-0533) [46]. This was further confirmed from the magnified curve in the appointed region (Figure S2a). Moreover, it was easily found that the peak intensity (26.5°) of CoO@G was much stronger than that of the G sample, which is mostly attributed to the Co-species introduced to improve the structure orderliness of carbon at a relatively high temperature to some extent. In contrast, there were no characteristic peaks at 26.5° in both Co@g-C_3_N_4_/G and g-C_3_N_4_/G samples. This phenomenon may be attributed to the fact that some of the g-C_3_N_4_ in the g-C_3_N_4_/G and Co@g-C_3_N_4_/G precursor under thermal treatment was partially decomposed [37,38], and the decomposed product could destroy the structure of carbon and result in an amorphous structure. Furthermore, the Co atoms could bind the N atoms to construct Co–N_x_ bonds [47,48], implanting the Co@g-C_3_N_4_/G material. Thereby, these mutual effects enhance the disorder degree of obtained materials. In addition, there were no obvious diffraction peaks of Co-species in the Co@g-C_3_N_4_/G sample compared to CoO/G. The differences may be ascribed to the highly dispersed ultrafine Co-species in the final product, and thus, it is hard to detect a characteristic signal, evidencing that the addition of g-C_3_N_4_ nanosheets could effectively alleviate the aggregation of Co^2+^ cations under an aqueous solution state. In addition, the annealing temperature as an influencing factor in the formation of the structure and composition of the obtained Co@g-C_3_N_4_/G products was further investigated. As displayed in Figure 1b, three samples almost exhibited similar XRD patterns at the corresponding temperature. However, an extra diffraction peak appeared at ~43.7° after calcination at 600 °C (red line in Figure 1b), corresponding to the (220) crystal plane of Co [46]. This result may be attributed to the heavy aggregation of metallic Co nanoparticles with the rise in the calcination temperature, thereby leading to the formation of large-size particles, displaying a distinct diffraction peak in the XRD pattern. Therefore, we could deduce that a minor feature of Co-species in the final products at a relatively low temperature was achieved, meanwhile giving rise to strong chemical adsorption/catalyzation conversion toward polysulfides due to enhanced surface energy and abundant active sites [29,31].

The Raman spectra of the three samples at different temperatures were also investigated, as displayed in Figure 1c. In each Raman spectrum, two apparent peaks could be seen at 1350 cm^−1^ and 1590 cm^−1^, corresponding to the D-band (sp^3^-hybridized carbon) and G-band (sp^2^-hybridized carbon) [10,25]. Generally, the intensity ratio of the D band to the G band (I_D_/I_G_) is used to evaluate the graphitization degree of carbon-based materials. The I_D_/I_G_ value significantly decreases with increasing calcination temperature, suggesting that a relatively high temperature could promote the graphitization of graphene.

The porous properties of the as-prepared samples were investigated using the N_2_ adsorption–desorption test at 77 K. The shape of the isotherms for the g-C_3_N_4_ and Co@g-C_3_N_4_/G samples were similar (Figure 1d and Figure S2b), that is, a certain amount of adsorption volume at a low relative pressure (*P/P*_0_ = 0.01–0.20), a H3 hysteresis loop at a medium relative pressure (*P/P*_0_ = 0.50–0.80), and an obvious uptake tendency at a high relative pressure (*P/P*_0_ = 0.90–1.0). These observations imply that the two prepared samples possessed a hierarchical porous structure, with the coexistence of micropores, mesopores, and some accumulated macropores [16,42], which were further identified from their pore size distribution curves. In addition, it was observed that Co@g-C_3_N_4_/G exhibited a tiny adsorption capacity at a low relative pressure (*P/P*_0_ = 0.01–0.20) compared to the g-C_3_N_4_ sample, indicating a relatively low specific surface area. This remarkable difference in these two samples may be due to the unique heterostructure of the Co@g-C_3_N_4_/G sample. The specific surface areas of g-C_3_N_4_/G and Co@g-C_3_N_4_/G were 124.4 and 48.73 m^2^ g^−1^. In addition, the pore size of Co@g-C_3_N_4_/G was predominately situated at meso-macropores regions, as can be seen from its pore size distribution curve (inserted in Figure 1d), which is conducive to promoting electrolyte penetration and Li ion transport in the cycling process [10]. 

**Figure 2:**
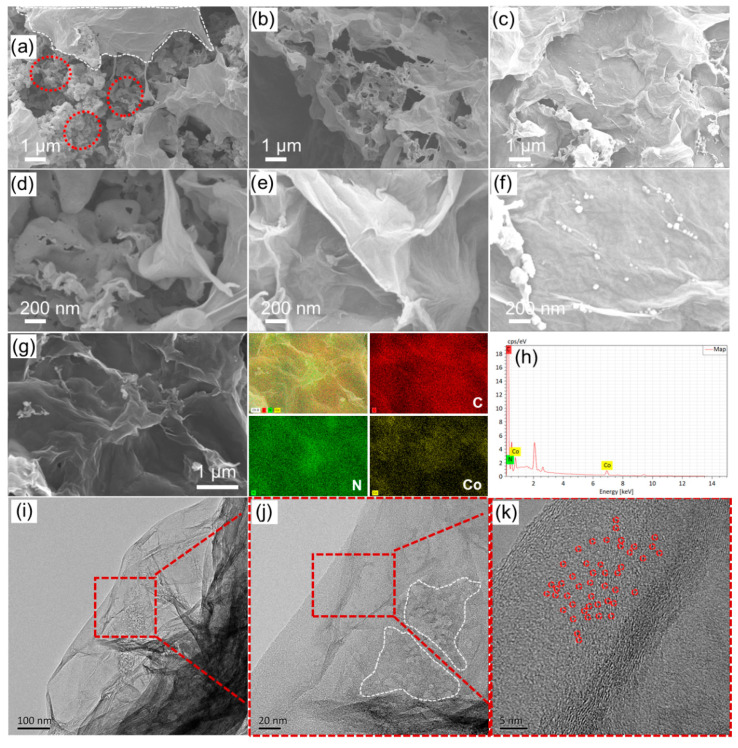
FE-SEM images of prepared Co@g-C_3_N_4_/G samples at different calcination temperatures: (**a,d**) 500, (**b,e**) 550, and (**c,f**) 600 °C. (**g,h**) SEM mapping and EDS of Co@g-C_3_N_4_/G at 550 °C. (**j**) TEM and (**i,k**) high–resolution TEM (HRTEM) images of the Co@g-C_3_N_4_/G sample.

The micromorphology of the corresponding samples was characterized using SEM and TEM, as shown in Figure 2. The SEM images of g-C_3_N_4_ and the precursor are displayed in Figure S3 (see Supporting Information). The g-C_3_N_4_ sample exhibited a flower-like scrolled structure with numerous crumpling and scrolling nanosheets (Figure S3a). As for the dried precursor, some flower-like scrolled g-C_3_N_4_ uniformly dispersed on the surface of wrinkled bulk graphene oxide nanosheets was clearly observed (Figure S3b). After calcination treatment at 500 °C, there was some flower-like scrolled g-C_3_N_4_ (marked in red circle) and large-size sheet-like substance (a typical feature of rGO marked in the white curve in Figure 2a,d), suggesting the coexistence of g-C_3_N_4_ and graphene. As the temperature increased to 550 °C, some crumpled, sheet-like structure was mainly displayed instead of the flower-like scrolled morphology. Specifically, numerous wrinkled, intersected nanosheets with a number of pores were observed in Figure 2b,e, which is mostly attributed to the partial decomposition of g-C_3_N_4_ with increasing temperature, thus generating a number of pores during the carbonization process [38]. Figure 2c,f also present a typical large-size crumpled, sheet-like structure from a macro point of view after annealing at 600 °C. Therefore, it could be concluded that g-C_3_N_4_ partially decomposes to realize the reduction of GO, meanwhile acting as a template to leave some pores in the calcination process. The microstructure of g-C_3_N_4_/G is similar to that of the Co@g-C_3_N_4_/G sample, as can be seen from the SEM images in Figure S3c,d. To further confirm the existence of elements in the prepared samples, the SEM mapping of Co@g-C_3_N_4_/G (550 °C) was performed, as shown in Figure 2g,h. In the SEM mapping of Co@g-C_3_N_4_/G, the elemental mapping images of a selective region of Co@g-C_3_N_4_/G exhibited a homogeneous dispersion of C, N, and Co, indicating these elements could be uniformly distributed on the surface of the sheet using this facile strategy.

The fine microstructure of Co@g-C_3_N_4_/G after calcination at 550 °C was further investigated using TEM and high-resolution TEM (HRTEM) images (Figure 2i–k). A stacked layer-like structure with some wrinkles and no agglomeration of Co species was clearly observed (Figure 2i), mostly corresponding to the typical rGO matrix. Specifically, Figure 2j displays a number of macro-/mesopores distributed in the framework (marked in white circles), which is conducive to an improvement in electrolyte penetration and Li ion diffusion according to the previous reported literature [15]. From the high-resolution TEM image (Figure 2k), numerous ultra-small-size Co-based nanodots (red circles) uniformly dispersed in a carbon framework were observed. This phenomenon may be due to the dual-anchoring effect of g-C_3_N_4_ and graphene oxide for Co^2+^ ions in the preparation of a precursor, effectively preventing the aggregation of Co-species in the calcination process. These ultra-small Co-based species can significantly catalyze the polysulfide conversion due to their high surface energy and more active terminations [30,36].

**Figure 3:**
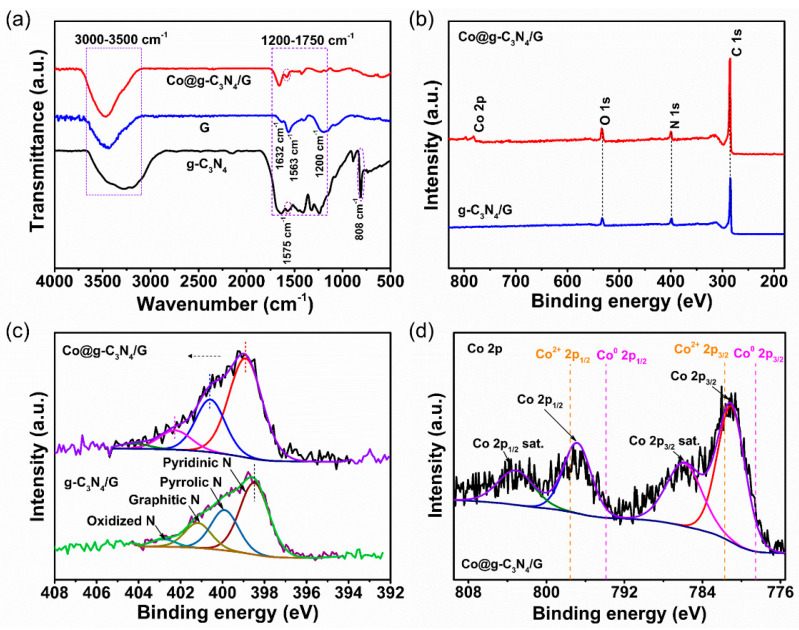
(**a**) FT−IR and (**b**) XPS survey spectra of the prepared samples. (**c**) Comparisons of N 1s high−resolution XPS spectra in Co@g-C_3_N_4_/G and g-C_3_N_4_/G samples. (**d**) Co 2p high−resolution XPS spectra in the Co@g-C_3_N_4_/G heterostructure.

Fourier-transform infrared spectroscopy (FT-IR) was conducted to investigate the chemical structures of the as-prepared composites (Figure 3a). The broad characteristic peaks located at ~3000–3500 cm^−1^ are indexed to the stretching vibrations of N-H from the marginal amino groups and O-H from the free adsorbed hydroxyl species [39,49]. Moreover, the adsorption peaks found at ~1200–1750 cm^−1^ for the g-C_3_N_4_ sample can be ascribed to the stretching vibrations of N-containing heterocycles [50]. Notably, a sharp peak was observed at ~808 cm^−1^ in g-C_3_N_4_, correlating with the typical tri-s-triazine ring vibration [39,50]. In the spectrum of the G sample, the three peaks at ~1632, 1565, and 1200 cm^−1^ corresponded to the stretching vibrations of C=O, C=C, and C-O, respectively [51]. For the Co@g-C_3_N_4_/G composites, most of the characteristic peaks of N-containing heterocycles generated from residual g-C_3_N_4_ were overlapped by graphene, while only a peak at ~1575 cm^−1^ referred to g-C_3_N_4_ still reappeared. These results indicate that some g-C_3_N_4_ in the Co@g-C_3_N_4_/G precursor under thermal treatment is partially decomposed, which is consistent with the previous result in [37,38]. To further determine the surface chemical state for the as-prepared samples, X-ray photoelectron spectroscopy (XPS) was performed. It was seen that two samples exhibited three distinct peaks at ~285.0, 399.1, and 533.1 eV (Figure 3b), corresponding to C 1s, N 1s, and O 1s, respectively. Furthermore, a Co 2p signal located at 781.5 eV was observed in the spectrum of the Co@g-C_3_N_4_/G sample. The corresponding atom ratios of the three samples (including the pure g-C_3_N_4_ originated from our recent work [42]) are listed in Table S1. Especially, the peak intensity of N 1s for the Co@g-C_3_N_4_/G and g-C_3_N_4_/G samples decreased sharply compared with that in the pure g-C_3_N_4_ sample (Figure S4). This significant difference is also recognized in Table S1, with the N atom ratios for the Co@g-C_3_N_4_/G (~7.12 at%), g-C_3_N_4_/G (~8.7 at%), and g-C_3_N_4_ (~75.02 at%) samples. According to other reported analogous g-C_3_N_4_/graphene composites [37,49], it can be further speculated that the added limited g-C_3_N_4_ in the precursor is decomposed partly under carbonization treatment. The N 1s XPS peak for the g-C_3_N_4_/G sample can be deconvoluted into four peaks (Figure 3c), which are indexed to pyridinic N (~398.5 eV), pyrrolic N (~399.9 eV), graphitic N (~401.2 eV), and oxidized N (~402.8 eV) [37,51], respectively. As for the Co@g-C_3_N_4_/G sample, all the fitted peaks for the high-resolution N 1s spectra had a slight shift toward higher binding energies, suggesting that some of the Co atoms in Co@g-C_3_N_4_/G bound with N atoms to form Co–N_x_ bonds [52,53]. It needs to be pointed out that these predominant pyridinic and pyrrolic nitrogen atoms have abundant chemisorption sites for polysulfides [16,25] and that the Co–N_x_ bonds can act as electrocatalytic centers for polysulfide adsorption/conversion [52,54], thus propelling the redox kinetics of sulfur species. The fitted high-resolution Co 2p spectrum in the Co@g-C_3_N_4_/G sample included four peaks (Figure 3d), which are assigned to the respective Co 2p_3/2_ (~781.1 eV), Co 2p_3/2_ satellite (~785.9 eV), Co 2p_1/2_ (~796.9 eV), and Co 2p_1/2_ satellite (~803.2 eV) [55]. Interestingly, the characteristic peaks of Co 2p_3/2_ (Co 2p_1/2_) are exactly situated between Co^0^ 2p_3/2_ (Co^0^ 2p_1/2_) and Co^2+^ 2p_3/2_ (Co^2+^ 2p_1/2_), indicating a cationic environment of Co species [54,55,56,57]. These results further reveal that a certain number of Co atoms in yjr Co@g-C_3_N_4_/G sample were prone to coordinating with N atoms to construct Co–N_x_ structures.

### 2.2. Visible Adsorption Experiments of Prepared Materials toward Li_2_S_6_ Solutions and Morphologies of g-C_3_N_4_/G- and Co@g-C_3_N_4_/G -PP Separators

**Figure 4:**
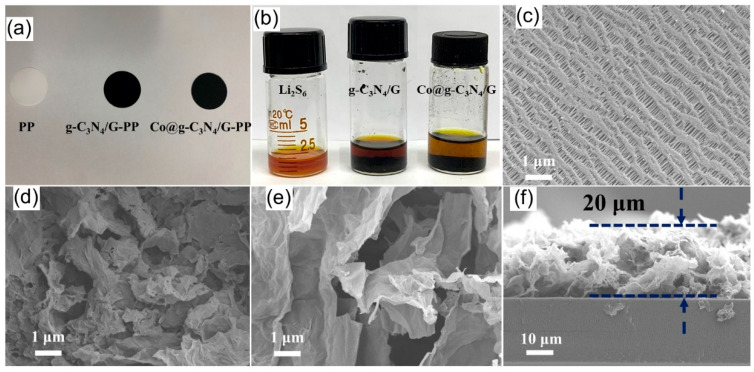
(**a**) Photographs of Li_2_S_6_ solution contacting with Co@g-C_3_N_4_/G and g-C_3_N_4_/G. (**b**) Photographs of PP, g-C_3_N_4_/G-PP, and Co@g-C_3_N_4_/G-PP separators. SEM images of (**c**) PP and (**d,e**) Co@g-C_3_N_4_/G-PP separators. (**f**) Cross-sectional SEM image of Co@g-C_3_N_4_/G-PP separators.

To better verify the capturing ability of Co@g-C_3_N_4_/G toward polysulfides, visible Li_2_S_6_ solution entrapment experiments were carried out and are shown in Figure 4a. First, host materials with the same mass (10 mg) were added independently to the prepared Li_2_S_6_ solution (2 mL) for static adsorption tests. The color of the Li_2_S_6_ solutions visibly changed from orange-yellow to slight yellow after standing for 6 h of adsorption in the bottle containing the Co@g-C_3_N_4_/G material. However, almost no color change in the bottle containing g-C_3_N_4_/G was observed compared to the blank Li_2_S_6_ solution. Even the color of Li_2_S_6_ solutions absorbed by the g-C_3_N_4_/G material looked rufous, which may be due to the mapping of black material at the bottom of the bottle. This phenomenon indicates that the Co@g-C_3_N_4_/G material has stronger adsorption ability toward polysulfides, further showing that Co@g-C_3_N_4_/G can effectively anchor intermediate polysulfide species by the synergistic effect of physical adsorption and chemical interaction [42,58]. Considering the unique affinity of Co@g-C_3_N_4_/G toward polysulfides, the Co@g-C_3_N_4_/G material was then used to modify the conventional PP separator for the improvement of electrochemical performance in Li-S cells. The modified separators were obtained using the vacuum-filtering method. In the optical images of functional separators using different coated materials (Figure 4b), an integrated modification layer without any detectable defect was seen, indicating the modified materials adhered well to the surface of the fresh separator. Scanning electron microscopy (SEM) was performed to further characterize the surface and cross-section morphologies of the modification layer. Evidently, the surface of the conventional PP separator in Figure 4c showed a typical macroporous feature, which easily led to the free diffusion of polysulfides across the separator. After introducing the Co@g-C_3_N_4_/G modification layer on the pristine PP separator, the macropores in the original PP separator were fully covered by the Co@g-C_3_N_4_/G material (Figure 4d). Moreover, this still maintained an obvious large-size sheet-like morphology of the predominate graphene in the coating layer, with some big voids between the interlaced lamellae, as shown in Figure 4e. This unique sheet-like structure can exactly act as a physical barrier to block the migration of polysulfides, and the interlaced voids are beneficial to electrolyte penetration and Li ion transport. In addition, the cross-sectional SEM image (Figure 4f) of the modified separator shows that the Co@g-C_3_N_4_/G interlayer attached well to the surface of the fresh PP separator, as well as having a thickness of ~20 um.

### 2.3. Electrochemical Performance of Li-S Batteries with the Modified Separators

**Figure 5:**
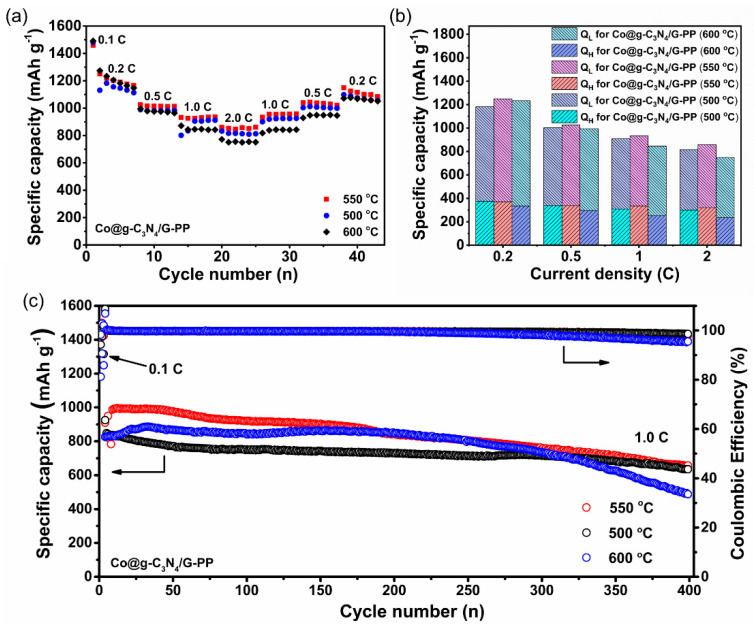
(**a**) Rate performances, (**b**) change in the high-plateau capacity and low-plateau capacity, and (**c**) long cycling performances of Li-S cells with the corresponding Co@g-C_3_N_4_/G-PP separators.

For the Co@g-C_3_N_4_/G material calcined at different temperatures, it was found that there was an obvious difference in the component and micromorphology based on our analyses. First, the performances of Li-S cells assembled with different Co@g-C_3_N_4_/G-PP separators were investigated. The electrochemical performances were tested using CR2016-type coin cells prepared by the corresponding separators and S/KB cathodes with a final sulfur content up to ~70 wt%. Figure 5a presents the rate properties of the corresponding Li-S cells recorded at various rates from 0.2 to 2.0 C. The cell using the Co@g-C_3_N_4_/G-PP (550 °C) separator delivered discharge-specific capacities of 1213, 1020, 935, and 860 mAh g^−1^ at a 0.2, 0.5, 1.0, and 2.0 C rate, respectively, suggesting an excellent rate property. Moreover, a reversible capacity of 1149 mAh g^−1^ was well recovered when the current density switched back to 0.2 C, demonstrating an effective confinement ability toward sulfur species at a high rate. In comparison, the cells with the other two functional separators exhibited inferior rate capabilities. Figure 5b and Figure S5 compare the voltage profiles of Li-S cells equipped with different separators under different current rates, and it can be observed that the cells with corresponding separators all retain a clear two-plateau discharge profile, even at a considerable rate of up to 2.0 C, but the capacities of both high- and low-voltage plateaus (Q_H_ and Q_L_) for the cell with the Co@g-C_3_N_4_/G-PP (550 °C) separator are much higher than those of the other two controlled cells, especially at faster rates, revealing an effective capture and catalytic conversion capability of the Co@g-C_3_N_4_/G (550 °C) interlayer toward polysulfide species [59,60]. Long cycling tests of cells assembled with corresponding separators were also performed (Figure 5c), and the cell with the Co@g-C_3_N_4_/G-PP (550 °C) separator exhibited the best cycling stability in comparison with the other two controlled cells. It delivered an initial specific capacity of 900 mAh g^−1^ and maintained a specific capacity of 657 mAh g^−1^ after 400 cycles at a 1.0 C rate, with a slow decline rate of 0.067% per cycle (capacity retention rate of 73.0%). In particular, a high and stable coulombic efficiency could be obtained concurrently in the whole cycling process, which indicates good electrochemical reversibility in the cell with the Co@g-C_3_N_4_/G-PP (550 °C) separator. Besides, it was seen that the cell with the Co@g-C_3_N_4_/G-PP (500 °C) separator delivered a relative low capacity, and another cell using the Co@g-C_3_N_4_/G-PP (600 °C) separator had severe capacity fading in later cycling (Figure 5c). Most possibly, the root reason for these results is a dual influence of electrical conductivity and Li ion transfer capacity in the Co@g-C_3_N_4_/G interlayer. Specifically, Co@g-C_3_N_4_/G possesses much more g-C_3_N_4_ and a relative low reduction degree of graphene after calcining at 500 °C, exhibiting limited electrical conductivity and thus leading to congested electronic transmission in the discharge–charge process [60]. As for Co@g-C_3_N_4_/G (600 °C), highly reduced graphene with obvious large-size sheets can hinder the transport of Li ions across the functional separator [61], resulting in the continuous accumulation of polysulfides over long-term cycling and then undergoing a rapid capacity decay. Hence, these observations indicate that the Co@g-C_3_N_4_/G (550 °C) material with superior electronic conductivity and Li-ion transfer capability is well fitted in separator modification for the better reuse of active sulfur. Given this, Co@g-C_3_N_4_/G (550 °C) as a separator modification material was adopted to compare with other controlled samples in the following electrochemical performance discussions. 

**Figure 6:**
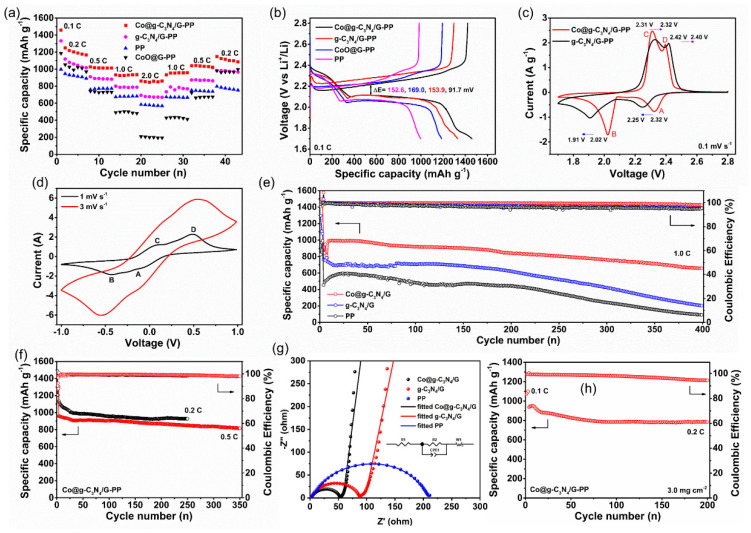
(**a**) Rate performances and (**b**) charge–discharge profiles at 0.1 C of Li-S cells with different separators. CV curves of (**c**) cells with g-C_3_N_4_/G-PP and Co@g-C_3_N_4_/G-PP separators and (**d**) symmetric cells using two Co@g-C_3_N_4_/G electrodes. (**e**,**f**) Cycling performances at different rates of cells with corresponding separators. (**g**) EIS spectra of cells with PP, g-C_3_N_4_/G-PP, and Co@g-C_3_N_4_/G-PP separators. (**h**) Cycling performance at 0.2 C of the cell using the Co@g-C_3_N_4_/G-PP separator with a high sulfur loading of 3.0 mg cm^−2^.

In this section, the whole obtained materials calcined at 550 °C were used to modify the PP separator for comparison of electrochemical performances in Li-S cells. As shown in Figure 6a, it was clearly observed that the cell with the Co@g-C_3_N_4_/G separator exhibited a superior rate property, delivering specific capacities of 1213, 1020, 935, and 860 mAh g^−1^ at 0.2, 0.5, 1.0, and 2.0 C, respectively, which are much higher than those of other cells with corresponding separators. As for the cell with the CoO/G-PP separator, it showed the worst rate performance, which is mostly due to the hindrance of the large-size sheet G coating layer to Li ion transfer and electrolyte penetration. This reason can be supported by other previously reported papers using graphene- and GO-functioned separators [16,23,62]. Figure 6b and Figure S5 show the representative charge–discharge profiles of cells with corresponding separators, all displaying two discharge plateaus and a long charging slope. Besides, we also calculated that the voltage hysteresis between charge and discharge in the four cells were 91.7, 153.9, 169.0, 152.6 mV, from the voltage curves in Figure 6b, revealing the smallest polarization in the cell with the Co@g-C_3_N_4_/G-PP separator. Further, this result can be confirmed from the CV curves in Figure 6c; two reduction peaks were observed at 2.32 (A) and 2.02 V (B) and two oxidation peaks were observed at 2.31 (C) and 2.42 V (D) for the cell with the Co@g-C_3_N_4_/G-PP separator, which exhibited a higher CV current and smaller polarization between peaks A and C compared to the control cell, meaning more enhanced redox reaction kinetics for polysulfide conversion in the charge–discharge process [25,59]. This is also supported by the EIS measurements (Figure 6g), the lowest charge transfer resistance (*R_ct_*: 49.4 Ω) for the cell with the Co@g-C_3_N_4_/G-PP separator among the three fresh cells, which is possibly ascribed to the improved catalytic conversion of polysulfides and the high electrical conductivity of the Co@g-C_3_N_4_/G interlayer [63]. To further determine the catalytic activity of the Co@g-C_3_N_4_/G heterostructure, Figure 6d shows the cyclic voltammetry (CV) curves of symmetrical cells assembled using two Co@g-C_3_N_4_/G-coated electrodes tested in the Li_2_S_6_ electrolyte. The CV curves of symmetric cells with respective electrodes all exhibited distinct peaks, which belonged to the reduction/oxidation of polysulfides. Since only Li_2_S_6_ served as the active material in this case, it was first reduced to short-chain polysulfides (peak A) and then Li_2_S (peak B) during the discharge process. In the subsequent charge process, Li_2_S was reversibly oxidized to polysulfides (peaks C and D) and the final sulfur element [64,65]. Four distinct redox peaks could be observed, even up to a scan rate of 3 mV s^−1^, in the Co@g-C_3_N_4_/G symmetrical cells, implying rapid kinetics conversion for polysulfide redox reactions in the Co@g-C_3_N_4_/G interface [25,66]. Therefore, these positive results confirm that the Co@g-C_3_N_4_/G heterostructure can well propel the kinetic conversion of intermediate polysulfides in a working Li-S cell. Figure 6e displays the cycling performances of the three cells with corresponding functional separators at 1.0 C, and it was obviously seen that the cell assembled with the Co@g-C_3_N_4_/G-PP separator exhibited the best cycling stability among the three cells, delivering the highest initial capacity of 900 mAh g^−1^ and still maintaining a specific capacity of 657 mAh g^−1^ after 400 cycles, with a capacity decay rate of ~0.067% per cycle and a high coulombic efficiency of above 98.0%. In addition, the cycling performance of cells with Co@g-C_3_N_4_/G-PP separators at low rates of 0.2 and 0.5 C were investigated, all exhibiting superior cycling stability, as shown in Figure 6f. There was moderate capacity fading in the first 30 cycles under the corresponding rates and then low capacity decay in the following appointed cycling numbers. Specifically, a specific capacity of 1040 mAh g^−1^ was delivered after a 0.1 C low rate activation and still remained at 928 mAh g^−1^ over 250 cycles at a 0.2 C rate, with a capacity-fading rate of 0.043% per cycle. At a 0.5 C rate, a stable capacity of 815 mAh g^−1^ was achieved after 350 cycles, with a low capacity attenuation of 0.035% per cycle. The comparison in Table S2 shows that the electrochemical performances of the cell with the Co@g-C_3_N_4_/G-PP separator have some advantages over other relevant functional-separator-assembled Li-S cells in the recent literature. Overall, these favorable results manifest a good promise of the as-designed separator in the improvement of the performance of Li-S batteries. Considering the superiority of the Co@g-C_3_N_4_/G-PP separators, a cell using the Co@g-C_3_N_4_/G-PP separator and a high-sulfur-loading S/KB cathode (3.0 mg cm^−2^) was prepared and its cycling stability was further explored (Figure 6h). The cell could deliver a high specific capacity of 1110 mAh g^−1^ at 0.1 C low rate activation and then undergo stable cycling at 0.2 C, still maintaining a capacity of 785 mAh g^−1^ after 150 cycles. These good electrochemical performances obtained can be attributed to two major aspects: (1) High-conductivity graphene can ensure fast electron transfer, which is conducive to accelerating the redox conversion of captured polysulfides occurring on the active sites of Co-species during the electrochemical process, and (2) the residual g-C_3_N_4_ supplies rich N sites for efficient adsorbing polysulfides, thus alleviating the shuttle effect and improving sulfur use. Hence, the lithium-sulfur batteries assembled with Co@g-C_3_N_4_/G-PP separators can possess a high capacity and stable cycling performance.

## 3. Materials and Methods 

### 3.1. Fabrication of Highly Dispersed Ultrafine Co-Species Interspersed g-C_3_N_4_ Nanosheets and Graphene Heterostructure (Co@g-C_3_N_4_/G)

g-C_3_N_4_ was prepared by direct thermal polymerization of urea in air, as referred from previous published work [41]. Co@g-C_3_N_4_/G heterostructure composites were synthesized via a one-step pyrolysis strategy. For preparation of the precursor, 25 mL of GO suspension (2.0 mg mL^−1^) was first sonicated and stirred for 0.5 h. Next, 50 mg of CoCl_2_·6H_2_O and the as-prepared g-C_3_N_4_ were added to this solution consecutively, followed by violent sonicating for another 2.0 h and vigorous stirring for another 1.0 h to produce a homogeneous mixture. Subsequently, the mixture was treated with vacuum filtration to remove the residual CoCl_2_·6H_2_O and then freeze-dried to obtain the precursor (g-C_3_N_4_/GO). Finally, the dry precursor was annealed under nitrogen protection and kept at different temperatures (500, 550, and 600 °C) for 2 h with a ramping rate of 5 °C min^−1^ to yield black products.

For comparison, a control sample of g-C_3_N_4_/G was prepared by following the same process without addition of CoCl_2_·6H_2_O. In addition, a CoO@G sample was obtained with pyrolysis of the precursor composed of CoCl_2_·6H_2_O and GO. Reduced GO (rGO) was obtained by direct thermal reduction of GO powder. These three controlled samples were all calcinated at 550 °C for 2 h with a heating rate of 5 °C min^−1^ under a nitrogen atmosphere.

### 3.2. Fabrication of the Co@g-C_3_N_4_/G-PP, g-C_3_N_4_/G-PP, and CoO@G-PP Separators

Modified separators were prepared using a vacuum filtration strategy. Briefly, 18.0 mg of Co@g-C_3_N_4_/G was dispersed into 150 mL of ethanol under ultrasonic treatment for 2 h, and then, 1.0 mL of the LA133 aqueous binder (0.2 wt%) was added to this solution to obtain a homogeneous slurry. Next, a certain volume of the prepared slurry was directly vacuum-filtered onto one surface of a commercial PP separator, followed by drying at room temperature, and finally punched into a 19-mm-diameter circular disk (Co@g-C_3_N_4_/G-PP). Similarly, the other corresponding separators were prepared using the same method. The areal mass density of the coating material was ~0.2 mg cm^−2^.

### 3.3. Electrochemical Measurements

The S/KB composite (the sulfur content in the S/KB composite was ~77 wt% displayed in Figure S1 [42]) was obtained through the conventional melt-diffusion method. Ketjen Black (KB: ECP600JD) and sulfur were uniformly ground in a weight ratio of 2:7 and then heated at 210 °C for 12 h in a closed autoclave filled with argon gas. The electrodes were prepared by casting the homogeneous slurry containing 90 wt% S/KB composites and 10 wt% PVDF binder onto a carbon-coated aluminum foil. The ordinary mass loading of sulfur in the as-prepared cathode was 1.3–1.5 mg cm^−2^. A high-sulfur-loading cathode (3.0 mg cm^−2^) was prepared by pasting the same slurry on aluminum foils. The electrochemical performances were tested using CR2025-type coin cells with different functional separators, assembled in an Ar-filled glove box with S/KB-based electrodes as cathodes and lithium foils as anodes. The electrolyte was composed of 1.0 M bis(trifluoromethanesulfony)imide lithium (LiTFSI) dissolved in a binary of 1,3-dioxolane (DOL) and 1,2-dimethoxyethane (DME) (1:1, *v*/*v*) containing 2 wt% LiNO_3_ additive. The electrolyte volumes were kept at 20 μL mg^−1^ sulfur for the ordinary-sulfur-loading electrodes and 12 μL mg^−1^ sulfur for the high-sulfur-loading electrodes.

Symmetric electrodes were prepared by pasting the homogeneous slurry containing Co@g-C_3_N_4_/G and PVDF binder in a weight ratio of 9:1 onto a circular carbon cloth (a diameter of 14 mm) with a mass loading of 0.5 mg cm^−2^. The catalytic conversion was evaluated using a symmetric battery, which was assembled using two identical tailor-made electrodes separated by a PP separator with 40 μL of 0.2 M Li_2_S_6_ electrolyte.

For the other tested methods, we referred to them from our recently reported papers (e.g., material characterizations, entrapment experiments, and various electrochemical tests) [25,42]. Additionally, it is pointed out that some of the physical characterization data of the pure g-C_3_N_4_ sample were from our recent work reported in [42].

## 4. Conclusions

In summary, g-C_3_N_4_ nanosheets and graphene decorated with the ultrafine Co-species heterostructure can act as a reinforced polysulfide barrier toward enhancing Li-S batteries. This modification layer can not only enable effective polysulfide affinity through the physical barrier and chemical binding but also simultaneously catalyze the conversion of intermediate polysulfides. As a result, Li-S cells assembled with Co@g-C_3_N_4_/G-PP separators matched with S/KB composites (up to ~70 wt% sulfur in the final cathode) exhibit excellent rate capability and good cyclic stability. A high specific capacity of ~860 mAh g^−1^ at 2.0 C as well as a capacity-fading rate of only ~0.035% per cycle over 350 cycles at 0.5 C can be achieved. This bifunctional separator can even endow a Li-S cell at a low current density to exhibit excellent cycling capability, with a capacity retention rate of ~88.4% at 0.2 C over 250 cycles. Furthermore, a Li-S cell with a Co@g-C_3_N_4_/G-PP separator possesses a stable specific capacity of 785 mAh g^−1^ at 0.2 C after 150 cycles and a superior capacity retention rate of ~84.6% with a high sulfur loading of ~3.0 mg cm^−2^. This effective polysulfide-confined separator holds good promise for promoting the further development of high-energy-density Li-S batteries.

## Data Availability

Data are available upon reasonable request.

## Supplementary Materials

The following supporting information can be downloaded at: https://www.mdpi.com/article/10.3390/molecules28020588/s1. Refs. [42,60,67,68,69,70,71,72,73,74] are cited in Supplementary Materials.
