# Supplementary material for: Ultrafine Co-Species Interspersed g-C3N4 Nanosheets and Graphene as an Efficient Polysulfide Barrier to Enable High Performance Li-S Batteries
Source: Molecules. 2023 Jan 6;28(2):588. doi: 10.3390/molecules28020588 (PMC9863667; doi:10.3390/molecules28020588)
Supplement: Supplementary file 1 [file molecules-28-00588-s001.zip › molecules-2081695-supplementary.pdf]

Ultrafine Co-species nanodots interspersed g-C_3N_4 nanosheets and graphene as an efficient polysulfides barrier to enable enhanced Li-S batteries

Shanxing Wang[1, 2], Xinye Liu[2], Yuanfu Deng[2, 3*]

[1] School of Chemistry and Materials Engineering, Huizhou University, Huizhou, 516007, China;

[2] The Key Laboratory of Fuel Cell Technology of Guangdong Province, School of Chemistry and Chemical Engineering, South China University of Technology, Guangzhou, China;

[3] Electrochemical Energy Engineering Research Center of Guangdong Province, South China University of Technology, Guangzhou, 510640, China;

E-mail address: chyfdeng@scut.edu.cn

Keywords: Co-species nanodots; g-C_3N_4 and graphene; Polysulfides barrier; Chemical adsorption; Catalytic conversion

Figures and Tables captions:

Figure S1. TG curve of S/KB composite.

Figure S2. (a) XRD pattern in the appointed region of CoO@G sample. (b) N_2 adsorption-desorption curve of pure g-C_3N_4.

Figure S3. FE-SEM images of (a) g-C_3N_4, (b) Co@g-C_3N_4/G precursor, (c, d) g-C_3N_4/G sample.

Figure S4. XPS survey spectra of pure g-C_3N_4 sample.

Figure S5. Charge-discharge profiles of Li-S cells with the Co@g-C_3N_4/G-PP based separators.

Table S1. Textural parameters of the corresponding samples.

Table S2. Electrochemical performance comparisons of Co@g-C_3N_4/G interlayer with other relevant interlayers in recent literatures.

Figure S1

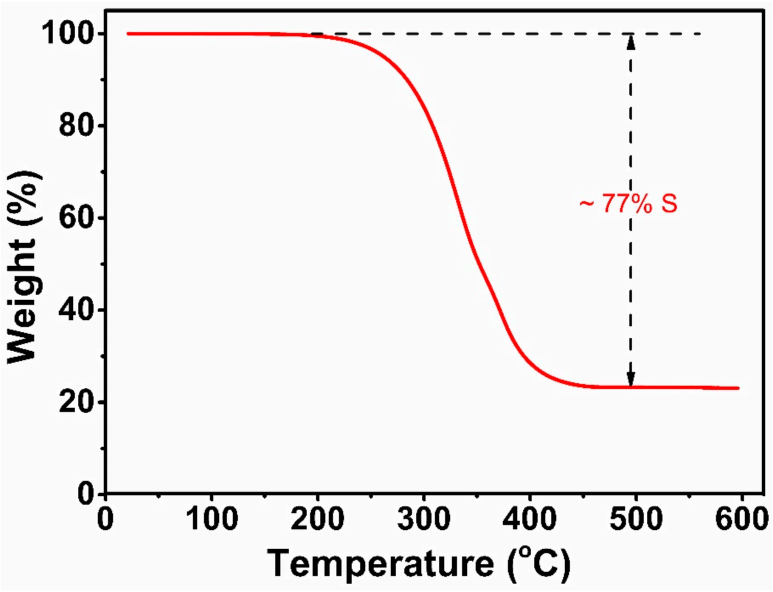

100
80
60
40
20
0
Weight (%)
0
100
200
300
400
500
600
Temperature (°C)
~ 77% S

Figure S2

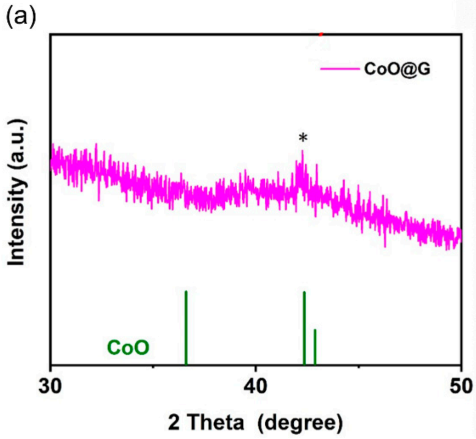

(a)
CoO@G
*
Intensity (a.u.)
CoO
30
40
50
2 Theta (degree)

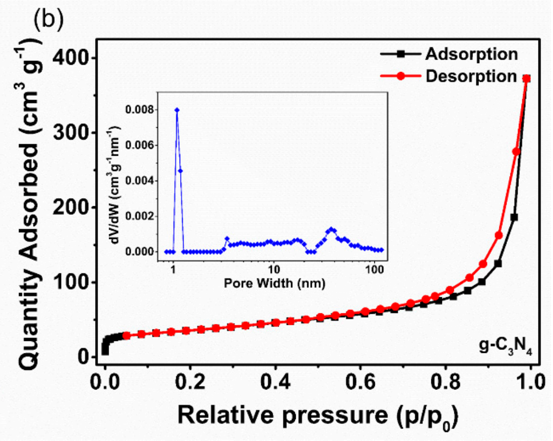

(b)
Adsorption
Desorption
Quantity Adsorbed (cm3 g-1)
400
300
200
100
0
dV/dW (cm3g-1nm-1)
0.008
0.006
0.004
0.002
0.000
1
10
100
Pore Width (nm)
g-C3N4
0.0
0.2
0.4
0.6
0.8
1.0
Relative pressure (p/p0)

Figure S3

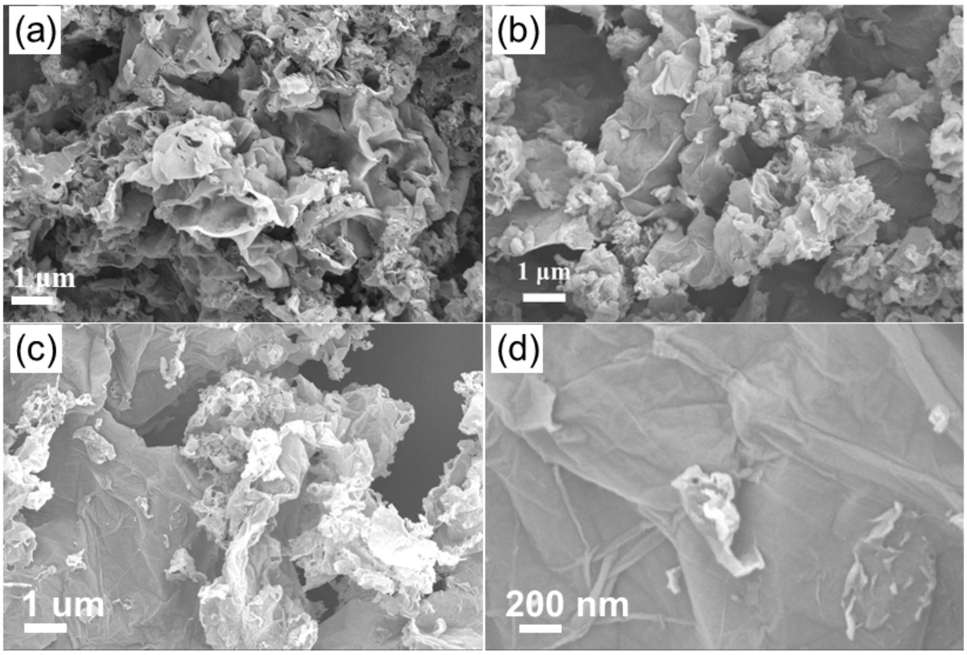

Figure S4

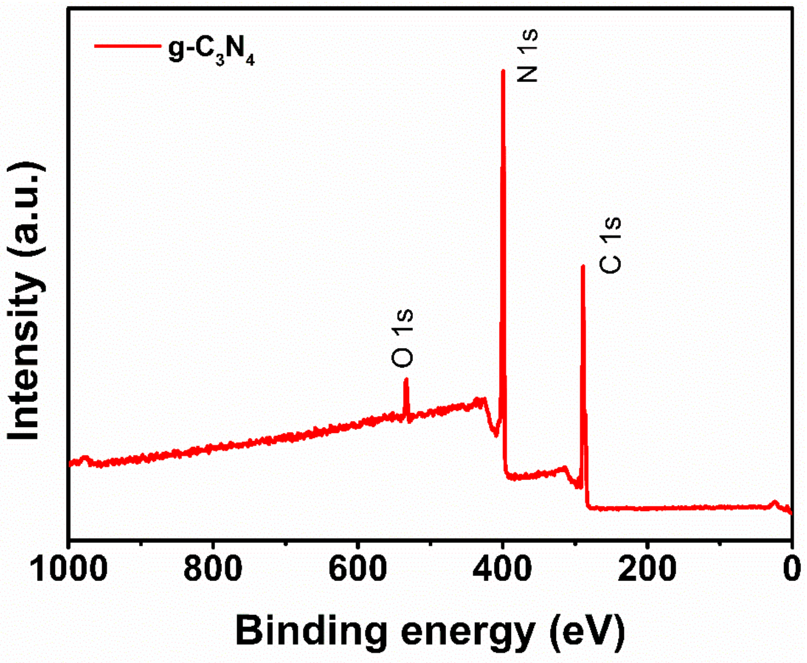
g-C3N4
N 1s
C 1s
O 1s
Intensity (a.u.)
1000
800
600
400
200
0
Binding energy (eV)

Figure S5

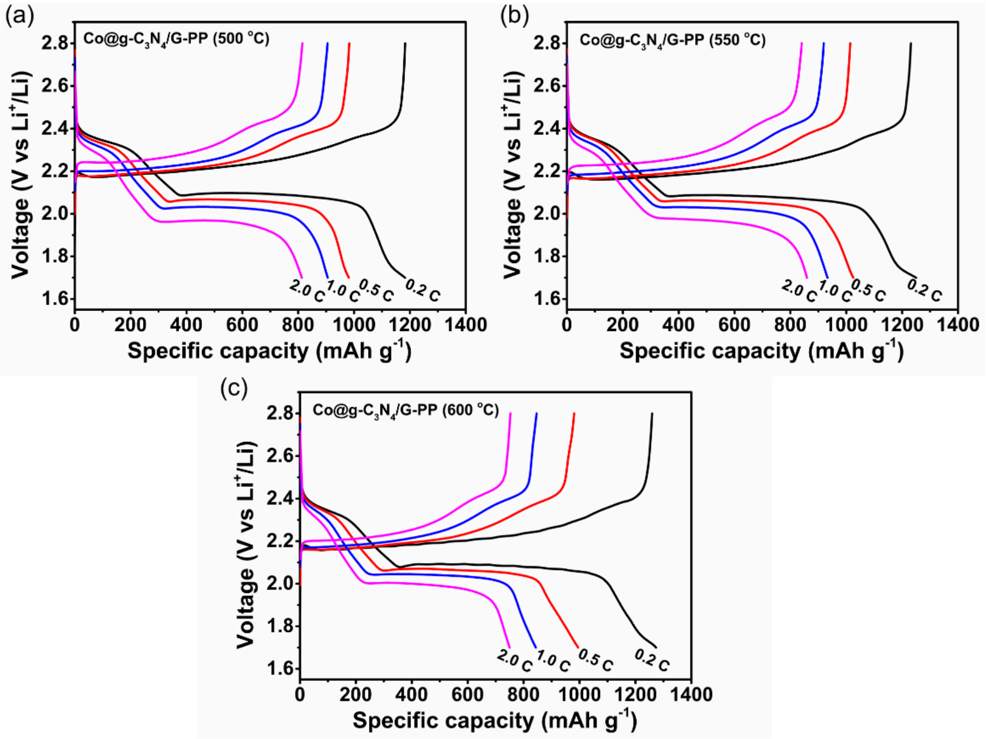

(a)
Co@g-C3N4/G-PP (500 °C)
Voltage (V vs Li+/Li)
Specific capacity (mAh g-1)
2.0 C
1.0 C
0.5 C
0.2 C
(b)
Co@g-C3N4/G-PP (550 °C)
Voltage (V vs Li+/Li)
Specific capacity (mAh g-1)
2.0 C
1.0 C
0.5 C
0.2 C
(c)
Co@g-C3N4/G-PP (600 °C)
Voltage (V vs Li+/Li)
Specific capacity (mAh g-1)
2.0 C
1.0 C
0.5 C
0.2 C

Table S1

Elements / Samples	C	N	O	Co
**g-C_3N_4**	18.94 %	75.02 %	6.04 %	–
**g-C_3N_4/G**	83.9 %	8.7 %	7.4 %	–
**Co@g-C_3N_4/G**	83.08 %	7.12 %	8.43%	1.37 %

Table S2

**Materials**	**Sulfur final content in cathode**	**Sulfur coating (mg/cm^2)**	**Interlayer coating (mg/cm^2)**	**Electrochemical performance (mAh/g)**
**Co@g-C_3N_4/G (This work)**	70 wt%	1.3-1.5	0.2	**785 after 100 cycles at 0.2 C (3.0 mg/cm^2)**<br>**657 after 400 cycles at 1.0 C (0.067 %)**<br>**815 after 350 cycles at 0.5 C (0.035 %)**<br>**928 after 250 cycles at 0.2 C (0.043 %)**
g-C_3N_4/CNT [S1]	60 wt%	1.4	0.45	870 after 100 cycles at 0.2 C (0.112 %)<br>800 after 500 cycles at 1.0 C (0.030 %)
G@g-C_3N_4/C/KB [S2]	72 wt%	1.0	0.35	770 after 100 cycles at 0.2 C (0.230 %)<br>475 after 800 cycles at 1.0 C (0.050 %)
$MnCo_2O_{4.5}$@g-C_3N_4/AB [S3]	63 wt%	1.3-1,4	-	791 after 100 cycles at 0.2 C (0.250 %)<br>632 after 400 cycles at 0.5 C (0.096 %)<br>553 after 500 cycles at 2.0 C (0.032 %)
Co-CN/Super P [S4]	60 wt%	1.0	0.3	640 after 250 cycles at 2.0 C (0.020 %)<br>719 after 100 cycles at 0.2 C (3.0 mg/cm^2)
g-C_3N_{4-x}/CNT [S5]	58.6 wt%	-	-	755 after 100 cycles at 0.2 C (0.310 %)<br>567 after 500 cycles at 1.0 C (0.067 %)
CoS@g-C_3N_4/KB [S6]	70 wt%	1.3-1.5	0.07	600 after 250 cycles at 0.1 C (4.0 mg/cm^2)<br>572 after 500 cycles at 1.0 C (0.03%)
Ni-C_3N_4/C [S7]	64 wt%	2.8	0.5	893 after 300 cycles at 0.3 C (0.035%)
FeOOH/C_3N_4/KB [S8]	64 wt%	1.2-1.5	0.6	444 after 900 cycles at 1.0 C (0.055 %)<br>570 after 500 cycles at 1.0 C (0.080 %)
N-C-Co film [S9]	60 wt%	1.5	0.24	660 after 250 cycles at 1.0 C (0.101 %)<br>735 after 250 cycles at 1.0 C (0.133 %
BCN/CB [S10]	52.5 wt%	1.2	0.5-0.6	553 after 500 cycles at 1.0 C (0.090 %)<br>760 after 300 cycles at 0.5 C (0.110 %

References

S1. Wang, X.L.; Li, G.R.; Li, M.J.; Liu, R.P.; Li, H.B.; Li, T.Y.; Sun, M.Z.; Deng, Y.R.; Feng, M.; Chen, Z.W. Reinforced polysulfide barrier by g-C_3N_4/CNT composite towards superior lithium-sulfur batteries. *J. Energy Chem.* **2021**, *53*, 234–240.

S2. Zhang, H.j.; Liu, Q.Z.; Ruan, S.J.; Ma, C.; Jia, X.F.; Qiao, W.M.; Ling, L.C.; Wang, J.T. In-situ construction of g-C_3N_4/carbon heterostructure on graphene nanosheet: An efficient polysulfide barrier for advanced lithium-sulfur batteries. *Appl. Surf. Sci.* **2022***, 578,* 152022.

S3. Sun, W.H.; Lu, Y.-C.; Huang, Y.Q. An effective sulfur conversion catalyst based on $MnCo_2O_{4.5}$ modified graphitized carbon nitride nanosheets for high-performance Li-S batteries. *J. Mater. Chem. A* **2021***, 9,* 21184–21196.

S4. Luo, M.; Bai, Y.; Sun, R.; Wang, Z.H.; Sun, W.; Lin, P.; Dai, X.; Sun, K.N. Enhanced performance of lithium-sulfur batteries with Co-doped g-C_3N_4 nanosheet-based separator. *Ind. Eng. Chem. Res.* **2021***, 60,* 1231−12405.

S5. Ma, H.; Song, C.L.; Liu, N.; Zhao, Y.; Bakenov, Z. Nitrogen-deficient graphitic carbon nitride/carbon nanotube as polysulfide barrier of high-performance lithium-sulfur batteries. *ChemElectroChem* **2020***, 7,* 4906−4912.

S6. Liu, X.Y.; Wang, S.X.; Duan, H.H.; Deng, Y.F.; Chen, G.H. A thin and multifunctional CoS@g-C_3N_4/Ketjen black interlayer deposited on polypropylene separator for boosting the performance of lithium-sulfur batteries. *J. Colloid Interf. Sci.* **2022**, *1*, 470−481.

S7. Chen, M.F.; Zhao, X.M.; Li, Y.F.; Zeng, P.; Liu, H.; Yu, H.; Wu, M.; Li, Z.H.; Shao, D.S.; Miao, C.Q.; Chen, G.R.; Shu, H.B.; Pei, Y.; Wang, X.Y. Kinetically elevated redox conversion of polysulfides of lithium-sulfur battery using a separator modified with transition metals coordinated g-C_3N_4 with carbon-conjugated. *Chem. Eng. J.* **2020**, *385*, 123905–123916.

S8. Wang ,Y.; Yang, L.W.; Chen, Y.X.; Li, Q.; Chen, C.T.; Zhong, B.H.; Guo, X.D.; Wu, Z.G.; Wang, G.K. Novel bifunctional separator with a self-assembled FeOOH/coated g-C_3N_4/KB bilayer in lithium-sulfur batteries. *ACS Appl. Mater. Inter.* **2020***, 12,* 57859−57869.

S9. Wang, J.N.; Wu, T.; Zhang, S.P.; Gu, S.; Jin, J.; Wen, Z.Y. Metal-organic-framework-derived N-C-

Co film as a shuttle-suppressing interlayer for lithium sulfur battery. *Chem. Eng. J.* **2018**, *334*, 2356.

S10. Jiang, R.J.; Jiang, M.; Huang, Z.Y.; Wang, J.; Kuang, Y.F.; Fu, C.P. Constructing light-weight polar boron-doped carbon nitride nanosheets with increased active sites and conductivity for high performance lithium-sulfur batteries. *Int. J. Hydrogen. Energ.* **2020**, *45*, 14940−14952.
